# Distances in Higher-Order Networks and the Metric Structure of Hypergraphs

**DOI:** 10.3390/e25060923

**Published:** 2023-06-12

**Authors:** Ekaterina Vasilyeva, Miguel Romance, Ivan Samoylenko, Kirill Kovalenko, Daniil Musatov, Andrey Mihailovich Raigorodskii, Stefano Boccaletti

**Affiliations:** 1 The Phystech School of Applied Mathematics and Computer Science, Moscow Institute of Physics and Technology, Institutskiy per., 9, 141701 Dolgoprudny, Moscow Region, Russia; 2 P.N. Lebedev Physical Institute of the Russian Academy of Sciences, Leninsky Prosp., 53, 119991 Moscow, Russia; 3 Departamento de Matemática Aplicada, Ciencia e Ingeniería de los Materiales y Tecnología Electrónica, Universidad Rey Juan Carlos, Calle Tulipán s/n, Móstoles, 28933 Madrid, Spain; 4 Mathematical Computation Laboratory on Complex Networks and Its Appliactions, Universidad Rey Juan Carlos, Calle Tulipán s/n, Móstoles, 28933 Madrid, Spain; 5 Faculty of Mathematics, National Research University Higher School of Economics, Usacheva str., 6, 119048 Moscow, Russia; 6 Scuola Superiore Meridionale, Largo S. Marcellino, 10, 80138 Napoli, NA, Italy; 7 Institute of Economics, Mathematics and Information Technology, Russian Academy of National Economy and Public Administration, pr. Vernadskogo, 84, 119606 Moscow, Russia; 8 Caucasus Mathematical Center, Adyghe State University, ul. Pervomaiskaya, 208, 385000 Maykop, The Republic of Adygea, Russia; 9 Mechanics and Mathematics Faculty, Moscow State University, Leninskie Gory, 1, 119991 Moscow, Russia; 10 Institute of Mathematics and Computer Science, Buryat State University, ul. Ranzhurova, 5, 670000 Ulan-Ude, The Republic of Buryatia, Russia; 11 CNR—Institute of Complex Systems, Via Madonna del Piano 10, 50019 Sesto Fiorentino, FI, Italy

**Keywords:** hypergraphs, higher-order networks, distances on hypergraphs, linegraphs

## Abstract

We explore the metric structure of networks with higher-order interactions and introduce a novel definition of distance for hypergraphs that extends the classic methods reported in the literature. The new metric incorporates two critical factors: (1) the inter-node distance within each hyperedge, and (2) the distance between hyperedges in the network. As such, it involves the computation of distances in a weighted line graph of the hypergraph. The approach is illustrated with several ad hoc synthetic hypergraphs, where the structural information unveiled by the novel metric is highlighted. Moreover, the method’s performance and effectiveness are shown through computations on large real-world hypergraphs, which indeed reveal new insights into the structural features of networks beyond pairwise interactions. Namely, using the new distance measure, we generalize the definitions of efficiency, closeness and betweenness centrality for the case of hypergraphs. Comparing the values of these generalized measures with their analogs calculated for the hypergraph clique projections, we show that our measures provide significantly different assessments on the characteristics (and roles) of the nodes from the information-transferability point of view. The difference is brighter for hypergraphs in which hyperedges of large sizes are frequent, and nodes relating to these hyperedges are rarely connected by other hyperedges of smaller sizes.

## 1. Introduction

The modeling of social, biological, and technological systems as complex networks (or graphs) has gained significant attention across areas of research as diverse as computer science, statistical physics, biology, neuroscience and social science, among others. Network science has, in fact, become ubiquitous [1]. In particular, as it was found that there is a deep interplay between the structural and dynamical features of networked systems, the analysis of the network topology therefore helps in understanding the network functional properties, such as robustness, efficiency in information transmission, and resiliency [1]. Metric parameters play a central role in deciphering information diffusion processes. Indeed, complex networks are renowned for their ability to efficiently transfer information or signal(s) from node to node; a proper way to quantitatively analyze the information flow is through the use of structural measures, such as the diameter, the characteristic path length, the closeness, the betweenness centralities, the efficiency, etc. [1,2,3].

The basis for the definition of all metric measures is the concept of *distance* between two nodes. If one deals with only dyadic node–node interactions, then there are no doubts as to how to define the distance between nodes, as the only way to construe a path from node *s* to node *t* is by means of a sequence of intersecting edges with the first one containing *s* and the last one containing *t*. Then, the corresponding path length is just the number of edges in the series, and the distance (or geodesic distance) between the two nodes is just the length of the shortest path.

However, the description of many processes in the real world requires to account for group interactions, i.e., interactions of more than two nodes, and it is, therefore, critical to refer to other mathematical objects that allow a better representation of such higher-order relationships [4,5]. These objects are called hypergraphs, and the quantification of efficiency of the spreading processes needs here the effort to extend the classical definitions of graph structural measures to such a hypergraph setting.

The simplest generalization to the higher-order case of any graph topological measure is just its computation in the hypergraph clique projection, a representation where each hyperedge is replaced by a clique of pairwise interactions among all its nodes. However, considering the clique projection, one obviously loses very important information about the structure of such higher-order interactions. This was brightly demonstrated in the series of recent studies, where it was shown that more complex generalizations of topological measures were needed in order to provide significant and meaningful information about the hypergraph structure [6,7,8,9,10,11]. In our study, we introduce a new concept of distance among nodes in a hypergraph.

Moreover, a series of fundamental macroscopic network topology descriptors (such as the betweenness centrality, the closeness centrality, or the efficiency) are explicitly based on the concept of metric structure or distance in the graph. As we will show further in more detail, the current literature contains some attempts to generalize the concept of distance [4,7,8,12,13,14], which, however, are not systematic, and are mostly only on one of the distinguishing features that higher-order structures display.

A first, naive extension for the term distance would be just replacing the word “edge” by the word “hyperedge” in the graph’s path definition given above, and leaving the rest of the definition unchanged. This way, one would obtain the so-called “distance in the hypergraph clique projection”. While such a definition frequently appears in the literature [15,16,17,18,19,20,21,22], it actually oversimplifies the rich structure of higher-order interactions. Indeed, in the case of a classic (unweighted) graph, all edges are roughly equivalent, as they all have the same cardinality (equal to two), and furthermore, all their intersections have the same cardinality (equal to one). This is not, however, the case of higher-order systems, where hyperedges may differ in their size and may intersect differently. Therefore, it seems reasonable that such extra information be somehow considered in the hypergraph distance definition.

**Figure 1:**
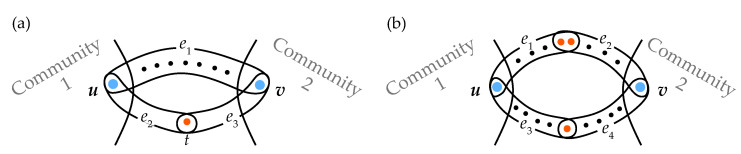
Illustrative sketch of two hypergraphs made of two communities of nodes, which are bridged by a series of hyperedges. Hypergraph in the panel (**a**) is used to provide explanation of the hyperedges’ cardinality importance for the path length calculation, while the one in the panel (**b**) illustrates the impact of hyperedges intersection size (see explanation in the text).

To make an illustrative example, let us refer to the two panels of Figure 1. In both cases, the hypergraphs are composed of two communities (of arbitrary internal structure) and contain hyperedges bridging the communities. Let us consider the hypergraph of Figure 1a, and let us suppose that a random walker starts at node *u* in the first community. Then, the probabilities to get from node *u* to node *v* by using no more than two steps either through the path including only the hyperedge $e_{1}$ ($p_{1}^{a}$) or by using the path $e_{2},\;e_{3}$ ($p_{2,3}^{a}$) are

$$\begin{array}{cc} p_{1}^{a} & =\underset{\underset{v\;\mathrm{is}\;\mathrm{chosen}\;\mathrm{randomly}\;\mathrm{on}\;\mathrm{the}\;\mathrm{first}\;\mathrm{step}}{⏟}}{\frac{1}{k_{u}}\cdot \frac{1}{|e_{1}|-1}\;}+\underset{\underset{\mathrm{some}\;\mathrm{other}\;\mathrm{node}\;\mathrm{from}\;e_{1}\;\mathrm{is}\;\mathrm{chosen}\;\mathrm{on}\;\mathrm{the}\;\mathrm{first}\;\mathrm{step}}{⏟}}{\frac{1}{k_{u}}\cdot \frac{|e_{1}|-2}{|e_{1}|-1}\cdot \frac{1}{|e_{1}|-1}\;}\;\;\;\;\;\;\;\;\;\;\;\;=\frac{1}{k_{u}}\cdot \frac{2|e_{1}|-3}{\left(\right|e_{1}{\left|-1\right)}^{2}},\\ p_{2,3}^{a} & =\frac{1}{k_{u}}\cdot \frac{1}{2}, \end{array}$$

where $|e_{1}|$ is the edge $e_{1}$’s cardinality (in Figure 1a $|e_{1}|=9$) and $k_{u}$ is the number of hyperedges incident to node *u*. It is clear that $p_{1}^{a}$ is significantly smaller than $p_{2,3}^{a}$, so it is natural to suppose that the length of the path $\left\{e_{1}\right\}$ should be larger than the length of the path $\left\{e_{2},e_{3}\right\}$.

Now, if one considers instead the hypergraph of Figure 1b and, as before, compares the probabilities $p_{1,2}^{b}$ with $p_{3,4}^{b}$ of getting from node *u* to node *v* by using no more than two steps, then one has

$$\begin{array}{cc} p_{1,2}^{b} & =\frac{1}{k_{u}}\cdot \frac{2}{5}\cdot \frac{1}{2}\cdot \frac{1}{5},\\ p_{3,4}^{b} & =\frac{1}{k_{u}}\cdot \frac{1}{5}\cdot \frac{1}{2}\cdot \frac{1}{5}. \end{array}$$

Now, one has $p_{1,2}^{b}=2p_{3,4}^{b}$, and therefore the path $\left\{e_{1},e_{2}\right\}$ should be assigned a distance shorter than that assigned to $\left\{e_{3},e_{4}\right\}$.

As a consequence, the concept of *length of a path* in a hypergraph should not only take into account the number of hyperedges involved but also their size and the size of their intersection: the bigger the size of a hyperedge, the larger the associated distance should be, and the bigger the intersection between hyperedges, the closer the nodes should be. Such two distinguishing features (varying sizes of hyperedges and sizes of hyperedges intersections) are indeed *separately* discussed in the literature, with the following in particular:

(1) If one assigns weights to hyperedges as proper functions of their sizes, then the distance between a couple of nodes s,t is the sum of the weights of all hyperedges in the shortest path, i.e.,
  (1) $$d\left(s,t\right)=\min_{P\in P_{st}}\left(\sum_{e\in P}f\left(|e|\right)\right),$$
  where $P_{st}$ is the set of paths between *s* and *t* and f(|e|) is the weight of the hyperedge *e* of size |e|. The heuristics behind this definition come from social networks: if one assumes that the size of each hyperedge is the size of a company of friends, then when the company size grows, it becomes more difficult for the members of the network to keep in touch with each other. This idea was used in Refs. [7,14], where the authors define hypergraph random walks, arguing that *“the walkers may give more or less importance to hyperedges depending on their size”*.
(2) The difference in the size of intersections between hyperedges was taken into account in the definition of *k*-walks introduced in Refs. [4,8,12,13]. A *k*-walk is a sequence of hyperedges such that each pair of successive hyperedges are adjacent, and they intersect in at least *k* vertices.

Despite the fact that both ingredients are separately considered in the structural analysis of hypergraphs, these two features have never, so far, been considered to be present together for the description of the metric structure of hypergraphs. The main goal of this paper is, therefore, to combine them in order to obtain a tailored metric structure for hypergraphs that would properly extend that of classic (dyadic) networks. Once this structure is defined (in the next section), network efficiency, closeness and betweenness centralities can also be defined as basic metric structural measures of the hypernetwork topology.

Our manuscript includes, therefore, three novel contributions to the literature. First, it introduces a new distance measure. Second, it gives the generalizations for the three crucial characteristics of network topology listed above. Third, it provides a series of examples showing the efficiency of the new concepts in uncovering meaningful information about higher-order networks. An illustration of such a novel approach is made with specific synthetic networks, where one immediately realizes that it provides important information on the properties of information transfer through the networks. Finally, our numerical testings on real-world higher-order networks reveal that the new metric measures uncover several important features of the hypergraphs properties. In particular, we show that our measures reveal different assessments of the nodes importance and of other network’s features from the information-transferability point of view. Finally, we show that our measure is particularly suitable to describe the structure of hypergraphs, which are highly distinguishable from graphs, i.e., in which hyperedges of large size are frequent and nodes in these hyperedges are rarely connected by other hyperedges of smaller sizes.

## 2. Methods

The basic notation used here is the same as that of Ref. [5]. In particular, a (dyadic) *complex network* is a (undirected and unweighted) network G=(V,E), where V={1,⋯,n} for some n∈N is the set of *nodes* (or vertices) and *E* is the set of *links* (or edges), i.e., a finite family of (unordered) pairs of nodes. Throughout this paper, only undirected and unweighted networks are considered, but all our results can be straightforwardly extended to the case of directed and weighted networks. A *hypergraph* is a pair H=(V,H), where V={1,⋯,n} is a (finite) set of nodes and $H=\{e_{1},\cdots ,e_{m}\}$ is a (finite) family of (non-empty) subsets of *V*, i.e., $e_{i}\subseteq V$ for all 1≤i≤m. Each element of *H* is called a *hyperedge* of H.

Notice that every complex network is a hypergraph, but the reverse is not true. Despite this latter fact, if one takes a hypergraph H=(V,H), one can define its complex network projection $\pi_{2}\left(H\right)=\left(V,\pi_{2}\left(H\right)\right)$, where each hyperedge $e_{i}\in H$ is replaced by the clique made of all the pairwise interactions among the hyperedge’s nodes, i.e., $\pi_{2}\left(H\right)=\left\{\left\{i,j\right\};\;i,j\in e_{k}\;\mathrm{for}\;\mathrm{every}\;e_{k}\in H\right\}$. $\pi_{2}\left(H\right)$ is called the *clique projection* of H.

In order to introduce a metric structure in H=(V,H) (i.e., a notion of distance between nodes of H), we first need to establish the notion of paths in H. If one takes a pair of nodes i,j∈V, a *path* from *i* to *j* is a (finite) sequence of hyperedges $({\tilde{e}}_{1},\cdots ,{\tilde{e}}_{k})\in H$ such that $i\in {\tilde{e}}_{1}$, $j\in {\tilde{e}}_{k}$ and ${\tilde{e}}_{\ell }\cap {\tilde{e}}_{\ell +1}\neq \emptyset$ for all 1≤ℓ<k. One can associate a (positive) real number to every path from *i* to *j* (the *length* of the path), and then the distance between *i* and *j* will be the minimal length among all the paths from *i* to *j*, so the critical point, as we will see in Section 2.1, is how to define the length for each path in a hypergraph.

The missing mathematical ingredient is the concept of the linegraph. Given a hypergraph H=(V,H), its (unweighted and undirected) *linegraph* L(H)=(H,L(H)) is a graph, whose node set is the set of hyperedges of H and for which there is a link between $e_{i},e_{j}\in H$ if $e_{i}\cap e_{j}\neq \emptyset$. Notice that the linegraph has self-loops in all its nodes since for every $e_{i}\in H$, one has that $e_{i}\cap e_{i}\neq \emptyset$. A weighted linegraph is introduced in Section 2.1 in order to define the path length in a hypergraph by assigning a non-negative weight to each link in L(H). Further properties and results about hypergraphs and their linegraphs can be found in Refs. [4,5].

### 2.1. Weighted Linegraphs

The use of weights in the links of the linegraph allows quantifying either the size of the hyperedges and that of the intersections among hyperedges. Given a hypergraph H, there is not a unique way of assigning weights to the links of its linegraph, so a weight distribution mechanism must be included. As we will show momentarily, the weights of the self-loops ($w_{ii}$) depend only on the size of corresponding hyperedge $e_{i}$, while the weights of the edges in the line graph are calculated as proper functions of the corresponding hyperedges’ sizes and the size of the intersection of these hyperedges.

**Figure 2:**
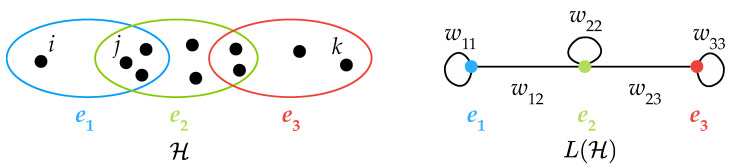
A 11-node and 3-hyperedge hypergraph H and its weighted line graph with self-loops L(H).

For the sake of illustration of our definitions, let us initially refer to the hypergraph (made of 11 nodes and 3 hyperedges) depicted in Figure 2, and its associated linegraph.

For every node i∈V of a hypergraph H=(V,H), one can denote by $E_{i}$ the set of hyperedges incident to the node *i* (in the example of Figure 2, $E_{i}=\left\{e_{1}\right\},\;E_{j}=\left\{e_{1},e_{2}\right\}$ and $E_{k}=\left\{e_{3}\right\}$). Then, one can define the *weighted distance* between the pair of nodes i≠k∈V as

(2) $$d^{w}\left(i,k\right)=1+\min_{e\in E_{i},\;\tilde{e}\in E_{k}}d^{L}\left(e,\tilde{e}\right),$$

where $d^{L}\left(e,\tilde{e}\right)$ is the distance between the hyperedges *e* and $\tilde{e}$ ($e\neq \tilde{e}$) in the weighted linegraph of H, while $d^{L}\left(e,e\right)$ is the weight of the self-loop {e,e} in the weighted linegraph. The critical point is now defining the weights of the linegraph in order to give sense to the definition given above.

**Figure 3:**
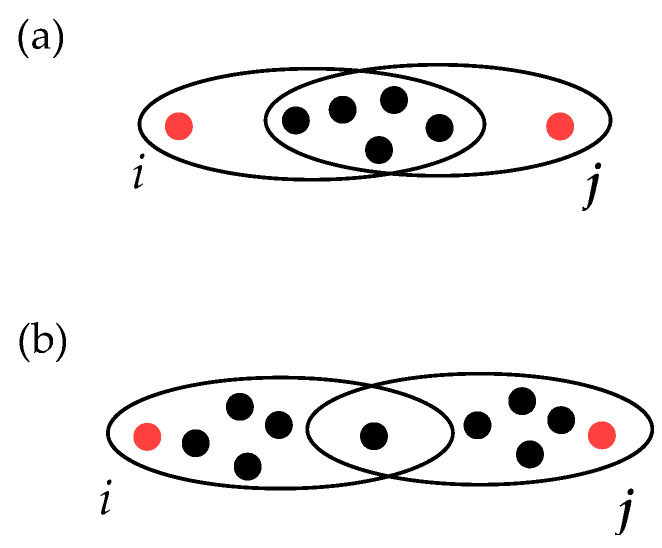
Two paths from nodes *i* to *j* in a hypergraph, each of them with 2 hyperedges of the same size but with different intersection size. The weighted path length in panel (**a**) should be smaller than that in panel (**b**).

**Figure 4:**
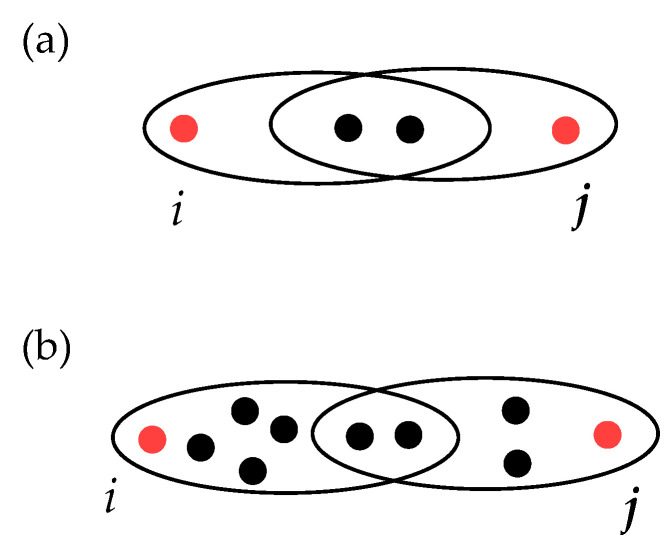
Two paths from nodes *i* to *j* in a hypergraph, each of them with two hyperedges with the same intersection size, but with different number of nodes in each hyperedge. The weighted path length in panel (**a**) should be smaller than that in panel (**b**).

**Figure 5:**
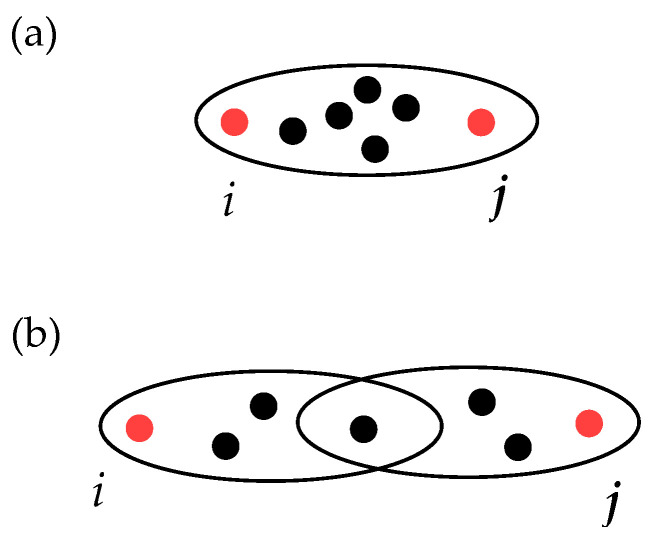
Two paths from nodes *i* to *j* in a hypergraph, one involving only one hyperedge and another with two hyperedges. Both paths include the same number of nodes. The weighted path length in panel (**a**) should be smaller than that in panel (**b**).

In particular, the weight function w:L(H)⟶[0,+∞) on the linegraph L(H)=(V,L(H)) must have the following properties:

1. The distance between the nodes obtained from Formula (2) must be the same as the classic distance in a graph at all times that one considers a (dyadic) complex network G=(V,E) as a hypergraph.
2. The bigger the intersection of the hyperedges, the smaller the distance should be. For instance, if one considers the case illustrated in Figure 3, then the weighted distance between *i* and *j* in panel (a) should be smaller than that in panel (b). Therefore, the distance should be inversely proportional to the intersection size.
3. The bigger the hyperedges involved, the larger the weighted distance should be. In particular, taking as an illustrative example the case of Figure 4, the weighted distance in panel (b) should be larger than that in panel (a) since the sizes of edges are bigger in case (b), while the size of the intersection is the same.
4. Finally, the larger is the number of hyperedges involved in the path, the larger weighed distances one should obtain. In other words, the new metric structure should be sensitive to the number of hyperedges in the paths considered. In particular, if one takes two paths, one with only one hyperedge and another with two hyperedges but with the same number of nodes involved in both cases, then the path length should be smaller in the first case (see the illustration in Figure 5, where panel (b) must give a larger distance with respect to the case of panel (a)).

A possible choice for the weight function w:L(H)⟶[0,+∞) that verifies the four desired properties is to assign, for every $\left(e_{i},\;e_{j}\right)\in L\left(H\right)$, a weight

(3) $$w_{ij}=w_{ji}=w(|e_{i}\cup e_{j}\left|,\right|e_{i}\cap e_{j}\left|\right)=\frac{1}{3}\left(|e_{i}\cup e_{j}|+\frac{|e_{i}\cup e_{j}|}{|e_{i}\cap e_{j}|}\right)-1.$$

It is easy, indeed, to verify that the function in Equation (3) accomplishes the following properties:

- As the Jaccard index [23] between $e_{i}$ and $e_{j}$ is given by $J\left(e_{i},e_{j}\right)=\frac{|e_{i}\cap e_{j}|}{|e_{i}\cup e_{j}|}$, then
  $$w_{ij}=\frac{1}{3}\left(|e_{i}\cup e_{j}|+\frac{1}{J(e_{i},e_{j})}\right)-1.$$
- If one takes $e_{i}=e_{j}$, then $w_{ii}=\frac{1}{3}\left(|e_{i}|+1\right)-1$. Furthermore, if one starts from a (dyadic) network G=(V,E), then $w_{ii}=0$ for every $e_{i}\in E$, and if $e_{i}\neq e_{j}$ (but $e_{i}$ connected with $e_{j}$ in *L*), then $|e_{i}\cup e_{j}|=3$ and $|e_{i}\cap e_{j}|=1$, which make that $w_{ij}=1$. Hence, one has that for this choice of weight function, the distance between the nodes obtained from formula (2) is the same as the classic distance in a graph (first desired property).
- Properties 2–4 also hold for this choice of weight function.

Let us discuss explicitly the issue of the computational complexity associated with the calculation of our distance. It is reasonable to analyze separately two steps of the distance calculations: the construction of the weighted linegraph and the distance calculation per se. As for the construction of the weighted linegraph, the worst-case estimation of the complexity is, obviously, $O(|H{|}^{2})$. Moreover, looking at Formula (2), estimating the distance $d^{w}\left(i,k\right)$ between nodes *i* and *k* needs the computation of the distances between all pairs of nodes $e\in E_{i}$ and $\tilde{e}\in E_{k}$ in the weighted linegraph L(H). The complexity associated with computing $d^{L}\left(e,\tilde{e}\right)$ is therefore given by the worst-case estimate for Dijkstra’s algorithm, which is O(|L(H)|+|H|log|H|) [24]. The resulting estimate of the computational complexity is $O(|E_{i}\left|\cdot \right|E_{k}\left|\cdot (|L\left(H\right)|+|H|\log|H|)\right)$.

To conclude this section, we remark that the choice of the weighting function in Equation (3) is not, in fact, unique, in the sense that other choices can be made that equally satisfy the four properties desired for a distance.

### 2.2. Some Structural Measures

The metric structure of a networked systems plays a central role in understanding the dynamics and processes that take place on them, including robustness [25], diffusion [1] and resilience [26]. There is a plethora of measures related to the metric structure of networks in the literature, and we will here limit ourselves to consider network efficiency, closeness and betweenness centrality, and to discuss their extension to the hypergraph setting by using the weighted distance introduced in Section 2.1.

The (global) efficiency of a graph, introduced in Ref. [27], measures the performance of the network information transfer. Formally, given a complex network G=(V,E) of *n* nodes, its efficiency is defined as

(4) $$E\left(G\right)=\frac{1}{n(n-1)}\sum_{i,j\in V,\;i\neq j}\frac{1}{d(i,j)},$$

where d(i,j) is the distance between nodes *i* and *j*.

It is straightforward to extend this measure for a hypergraph H=(V,H) of *n* nodes by simply considering the metric structure in H. As we discussed already in the introduction, one could consider a simple approach and define a metric structure in H given by its clique projection network $\pi_{2}\left(H\right)$. By this metric, one could define the efficiency of H as $E\left(H\right)=E(\pi_{2}\left(H\right))$. On the other hand, if one considers the weighted metric structure defined by Equation (2), then one can calculate the weighted efficiency of the hypergraph H as

(5) $$E^{w}\left(H\right)=\frac{1}{n(n-1)}\sum_{i,j\in V,\;i\neq j}\frac{1}{d^{w}\left(i,j\right)}.$$

Similarly, one can introduce a weighted hypergraph analogue of closeness centrality. If one takes again a network G=(V,E), then the closeness centrality [1,2,3] of node i∈V is

(6) $$C_{i}\left(G\right)=\frac{1}{\sum_{j\in V,j\neq i}d\left(i,j\right)}.$$

Notice that the top nodes (according to the closeness centrality values) can be seen as the most aware nodes in an information/social network [1,2,3]. By using expression (6), it is easy to transfer this concept to the hypergraph setting, simply by computing

(7) $$C_{i}^{w}\left(H\right)=\frac{1}{\sum_{j\in V,j\neq i}d^{w}\left(i,j\right)},$$

for every node *i* in a hypergraph H=(V,H). Additionally, in this case, if one considers the metric structure given by the clique projection $\pi_{2}\left(H\right)$, then one can define an alternative closeness centrality as $C_{i}\left(H\right)=C_{i}\left(\pi_{2}\left(H\right)\right)$ for every node *i* in the hypergraph.

Finally, the weighted centrality of a node *i* in a hypergraph H=(V,H) can also be defined, but in this case, one should pay attention to some extra remarks. Let us remind indeed that, if one takes a network G=(V,E), the betweenness centrality of each node *i* is given by

(8) $$b_{i}\left(G\right)=\sum_{u,v\in V,u\neq v,u\neq i,v\neq i}\frac{\left|P(u,v,i)\right|}{\left|P(u,v)\right|},$$

where P(u,v) is the set of all shortest paths between *u* and *v*, whereas P(u,v,i) is the set of shortest paths between *u* and *v* that pass through *i* and |·| is the set cardinality operator, i.e., it is the sum of fractions of shortest paths between all possible nodes pairs in the graph going through node *i*.

If one wants to extend this notion to a general hypergraph H=(V,H), one should specify first what it means that a path from *u* to *v* goes through node *t* when the path is a series of hyperedges. Following Ref. [18], we assume that if we have a path described by the sequence of hyperedges $\left\{e_{1},\ldots ,e_{\ell }\right\}$, where $u\in e_{1}$ and $v\in e_{\ell }$, then it provides us with a sequence of nodes $\left\{u,t_{1},t_{2},\ldots ,t_{l-1},v\right\}$, where $t_{i}\in e_{i}\cap e_{i+1}$ (nodes $t_{i},\;i=1,\ldots ,l-1$ are called *bridging nodes* in Ref. [18]). Once we fix this notion, the weighted betweenness centrality of a node *i* in a hypergraph H=(V,H) is defined as

(9) $$b_{i}^{w}\left(H\right)=\sum_{u,v\in V,u\neq v,u\neq i,v\neq i}\frac{\sum_{p\in P_{e}^{w}\left(u,v,i\right)}\frac{1}{int_{i}\left(p\right)}}{|P_{e}^{w}\left(u,v\right)|},$$

where $P_{e}^{w}\left(u,v\right)$ is the set of shortest paths between nodes *u* and *v* whose lengths are calculated by using the proposed weighted hypergraph measure, and the elements of the set are all possible sequences of hyperedges forming a path from node *u* to node *v*; $P_{e}^{w}\left(u,v,i\right)$ is the set of those shortest paths between nodes *u* and *v*, for which the intersection of one pair (or some pairs) of hyperedges includes node *i*; and $int_{i}\left(p\right)$ is the size of such a latter intersection. Once again, if one considers the clique projection $\pi_{2}\left(H\right)$, then one could define an alternative betweenness centrality as $b_{i}\left(H\right)=b_{i}\left(\pi_{2}\left(H\right)\right)$ for every node *i* in the hypergraph.

## 3. Results

Let us compare the new weighted metric structure with the classic approach based on the clique projection with reference to a series of illustrative synthetic hypergraphs and with large-sized real-world higher-order networks.

### 3.1. Synthetic Examples

**Figure 6:**
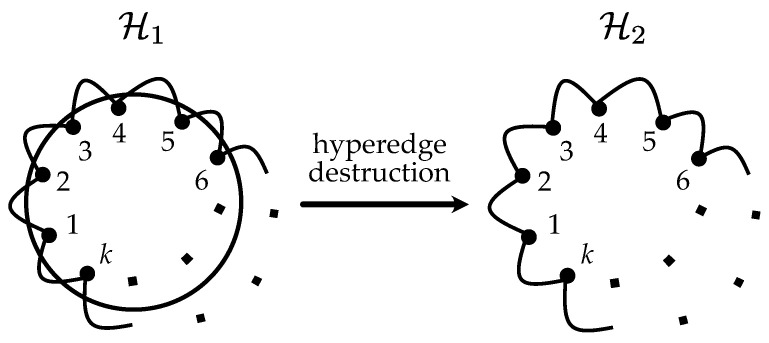
An illustrative example of a ring-like hypernetwork with an additional hyperedge grouping all nodes. If the central hyperedge is removed, the hypernetwork is transformed into a ring structure.

In the first example, we consider the hypernetwork $H_{1}$ described in Figure 6. The hypergraph contains *k* nodes organized in the form of a ring, with an additional hyperedge grouping all nodes. Let us assume that hyperedges may be eliminated with a probability proportional to their size, an assumption which is natural in social networks since it is hard to maintain communication within huge teams. Considering this process, in our example, one has, therefore, a very high probability to obtain graph $H_{2}$ (the right panel of Figure 6), which consists of just a ring structure without a central hyperedge.

Table 1 shows the efficiency values calculated by using the proposed weighted hypergraph distance ($E^{w}\left(\cdot \right)$) and the traditional one, based on the clique projection (E(·)). One immediately sees that the traditional measure overestimates the hypergraph robustness, whereas the fact that the central hyper-link is not reliable is correctly reflected in the case of our distance measure. This conclusion follows immediately from a comparison of the $E(H_{1})$ and $E(H_{2})$: in the case of the new distance definition, $H_{1}$ and $H_{2}$ have approximately the same efficiency, while in the case of the traditional measure, efficiency drops significantly when we destroy the central hyperedge.

**Figure 7:**
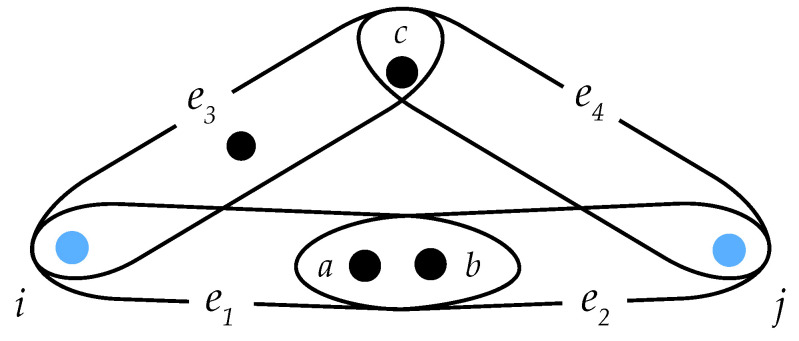
Illustrative example of a hypergraph, where information transfer occurs from a source node *i* to a target node *j*. The hypothesis is made that in each intermediate node, information is copied and errors may appear. In the example, there are two possible paths from node *i* to node *j*: $\left(e_{1},e_{2}\right)$ and $\left(e_{3},e_{4}\right)$. a,b,c are nodes forming the intersection between different hyperedges.

In the second example, we center our attention on the hypergraph of Figure 7. There, the assumption is made that one needs to transfer information from a source node *i* to a target node *j*, and in each intermediate node, information is copied and errors may appear. Therefore, the smaller the number of intermediate nodes, the less contaminated by errors the information will arrive at the target node. In addition, one assumes that the nodes of the hypernetwork can be attacked, and therefore some paths can be eliminated, which makes it such that the higher the intersection size is, the more reliable the communication.

Let us now consider two existing paths from node *i* to node *j*: $\left(e_{1},e_{2}\right)$ and $\left(e_{3},e_{4}\right)$. It is straightforward to check that these two paths are equivalent in the classic metric structure obtained from the clique projection. However, the first path is shorter than the second by using the weighted distance since the size of the edges union is the same for the both paths, while the intersection size is higher in the first case.

### 3.2. Real World Examples

We concentrate on the analysis of three real-world hypergraphs taken from https://www.cs.cornell.edu/~arb/data/ (accessed on 11 June 2023). The first is the Contact High School hypergraph [28,29], where nodes are students and hyperedges represent communications between them. The second hypernetwork is the Email Enron dataset based on lists of e-mails’ senders and recipients (only hyperedges with a size up to 25 are considered, following Ref. [28]). The third hypergraph is reconstructed from Senate Committees data [10,30], where nodes are members of the US Senate, and hyperedges correspond to committee memberships.

The general characteristics and parameters of the three considered datasets are reported in Table 2, where it is shown that such hypergraphs have a comparable number of nodes, but they are significantly different in the properties of their hyperedge size distributions. In particular, the Contact High School hypergraph is characterized by the smallest size of the largest hyperedge. Furthermore, despite the fact that in the Email Enron data, one has significantly larger hyperlinks than in the Contact High School one, the median hyperedge size in both hypergraphs is equal to two, and the mean hyperedge size in Email Enron is also not far from the one characterizing the Contact High School case. The Senate Committees hypergraph differs from the other two in all the hyperedges distribution characteristics, and for this latter hypergraph, one has a relatively small number of hyperedges, which are, however, large.

**Figure 8:**
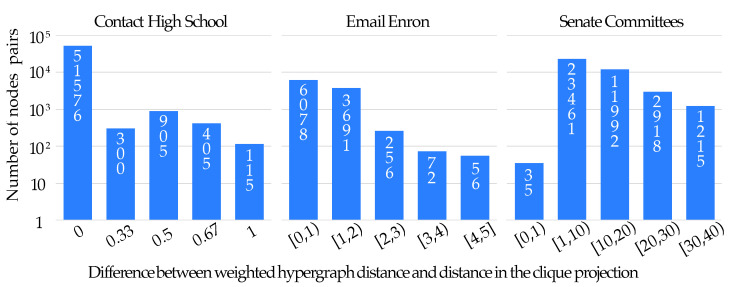
Distributions of differences between the proposed weighted hypergraph distance measure and the one calculated in the clique projection approach, for the real world hypergraphs analyzed in our study. In the first histogram, the number of pair of nodes is reported for which the difference between the two calculated distances takes the values specified in the horizontal axis. In the second and third histograms, we report instead the number of pairs of nodes, for which the difference between the two distances lies within the intervals indicated in the horizontal axis.

In order to point out the additional information provided by the proposed weighted distance measure, one can calculate (and compare) the distances between all pairs of nodes in the considered hypergraphs by using our measure and the clique projection approach. The distributions of the differences between the results obtained with our measure and those obtained with the clique projection are presented in Figure 8. From these diagrams, one clearly sees that in the Contact High School data, the difference is not significant. Therefore, one can conclude that in this hypergraph, there is a large number of relatively short paths going only through edges of size two, alternative to the paths including edges of larger sizes. This conclusion is, however, incorrect for the other two hypernetworks, especially for the Senate Committees one. One sees, indeed, that the distance in the clique projections of the hypergraphs is significantly smaller then the weighted one. Therefore, one expects a higher vulnerability of these latter graphs with respect to large edge removal.

If one wants to give a more quantitative ground to the last remark, one can calculate for each of the hypergraphs two efficiency measures: the one using the proposed weighted distance ($E^{w}\left(\cdot \right)$) and the other based on traditional distance measure (E(·)). The results are shown in Table 3, where it is shown that the estimates have close values for the Contact High School hypergraph, whereas the values are significantly different for Email Enron and Senate Committees. If one compares the hypernetwork efficiencies using the traditional measure, one should conclude that the most efficient hypergraph is the Senate Committees one. On the contrary, by using the proposed weighted distance, one actually is led to the opposite conclusion. Notice that if our hypothesis that in social networks larger groups are less stable is correct, then only the $E^{w}\left(\cdot \right)$ measure provides a correct comparison of the hypergraph efficiencies.

Besides efficiency, one can analyze node rankings based on the closeness and betweenness centralities by using either the traditional or the weighted hypergraph distances, as described in Section 2.2. In order to compare the rankings, one can use the Kendall rank correlation (KRC) and a measure μ that quantifies correlation given by

(10) $$\mu_{N}\left(x,y\right)=\frac{|{\mathrm{top}}_{N}\left(x\right)\cap {\mathrm{top}}_{N}\left(y\right)|}{N},$$

where *N* is the number of top nodes considered, x and y are the two rankings, and ${\mathrm{top}}_{N}\left(x\right)$ is the set of the *N* top nodes in the ranking x. In particular, we compare rankings $b={\left(b_{1}\left(H\right),\ldots ,b_{N}\left(H\right)\right)}^{\prime }$ and $b^{w}={\left(b_{1}^{w}\left(H\right),\ldots ,b_{N}^{w}\left(H\right)\right)}^{\prime }$ obtained with the betweenness centrality, and rankings $C={\left(C_{1}\left(H\right),\ldots ,C_{N}\left(H\right)\right)}^{\prime }$ and $C^{w}={\left(C_{1}^{w}\left(H\right),\ldots ,C_{N}^{w}\left(H\right)\right)}^{\prime }$ obtained with the closeness centrality for the three real hypergraphs.

**Figure 9:**
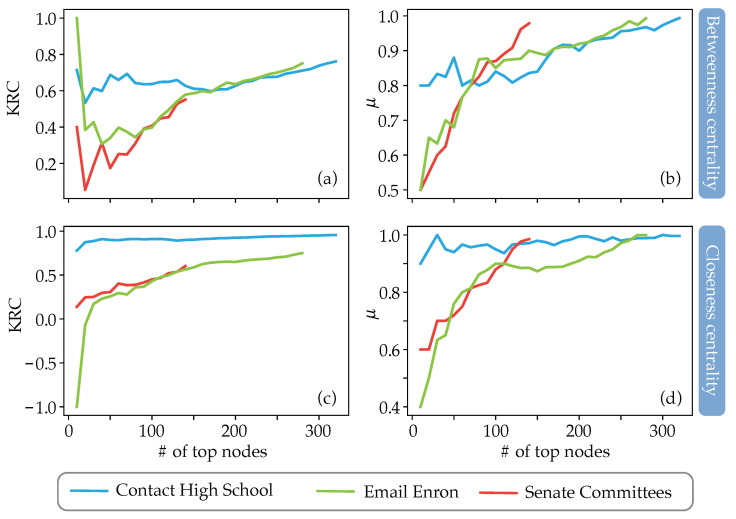
Correlations between the rankings based on betweenness (panels (**a**,**b**)) and closeness (panels (**c**,**d**)) centralities, calculated using the traditional distance measure and the weighted hypergraph one. (**a**,**c**) Kendall rank correlation (KRC) coefficients between the top nodes rankings. (**b**,**d**) The μ measure values (see Equation (10) for the definition of the μ measure). In all panels, the horizontal axis reports the number of top nodes considered in the rankings. The color code for identifying the curves plotted in all panels is reported in the horizontal bar at the bottom of the figure.

The results are presented in Figure 9, and the following conclusions can be drawn:

1. In the Contact High School hypergraph, the difference in rankings is significantly less considerable than that occurring in the other two hypergraphs, especially for the case of closeness centrality;
2. The sets of the first 50 top nodes for the cases of the Email Enron and Senate Committees hypergraphs are significantly different when one uses the traditional distance measure and our measure for both betweenness and closeness centralities (see Figure 9b,d);
3. The KRC coefficient is low in the case of betweenness centralities, even for the case of the Contact High School hypergraph (see Figure 9a);
4. The KRC coefficient for the case of closeness centrality is low for the Email Enron and Senate Committees hypergraphs (see Figure 9c), being even negative for the case of the Email Enron graph.

Summarizing the above evidence, one can say that the proposed distance measure provides significantly different assessments on the characteristics (and roles) of the nodes from the information-transferability point of view. The difference is brighter for the hypergraphs which are principally distinguished from graphs, i.e., in which hyperedges of large size are frequent and nodes in these hyperedges are rarely connected by other hyperedges of smaller sizes.

## 4. Conclusions

In conclusion, we here introduced a novel definition of distance for hypergraphs that extends the classic methods reported in the literature. In particular, the new distance takes explicitly into account two critical factors (the inter-node distance within each hyperedge and the distance between hyperedges in the network) which have never, so far, been considered together. The consequence is that the computation of distances is made in a properly weighted linegraph of the original hypergraph. We illustrated the benefit of adopting such a metric structure with reference to a series of small synthetic hypernetworks, and we then applied our approach to large real-world hypergraphs, revealing that the obtained information about the hypergraphs structure is far different from that which is acquired by computing distances with the standard approach of clique projection. In particular, the difference is significant for hypergraphs in which hyperedges of large sizes are frequent and nodes are rarely connected by hyperedges of small sizes. This latter evidence points to the fact that our measure may be of great help in various circumstances, especially when a correct assessment of the roles of nodes is needed from the information-transferability point of view.

Possible follow-ups of our study include the use of our novel definition of distance for the measure of other topological properties of (or the analysis of processes taking places in) hypergraphs. This is the case, for instance, for random walks, local efficiency, clustering coefficients, community structure features, modularity, etc. Furthermore, a similar approach can be adopted also in the case of directed and annotated hypergraphs.

## Figures and Tables

**Table 1:** Efficiency values of $H_{1}$ and $H_{2}$ calculated using different distance definitions. k=20.

	$E^{w}\left(\cdot \right)$	E(·)
$H_{1}$	0.311	1
$H_{2}$	0.303	0.303

**Table 2:** General characteristics of the real-world hypernetworks analyzed in our study.

	High School	Email Enron	Senate Committees
Number of nodes	327	143	282
Number of unique hyperedges	7818	1457	315
Maximal hyperedge size	5	18	31
Minimal hyperedge size	2	2	4
Mean hyperedge size	2.3	3.1	17.2
Median hyperedge size	2	2	19

**Table 3:** Efficiency of the real-world hypergraphs computed using the proposed weighted hypergraph distance measure ($E^{w}\left(\cdot \right)$) and the traditional distance measure (E(·)).

	Contact High School	Email Enron	Senate Committees
$E^{w}\left(\cdot \right)$	0.505	0.443	0.106
E(·)	0.510	0.546	0.670

## Data Availability

Publicly available datasets were analyzed in this study. This data can be found here: https://www.cs.cornell.edu/~arb/data/ (accessed on 11 June 2023).
